# Safety and feasibility of minimally invasive gastrectomy during the early introduction in the Netherlands: short-term oncological outcomes comparable to open gastrectomy

**DOI:** 10.1007/s10120-017-0695-8

**Published:** 2017-02-09

**Authors:** H. J. F. Brenkman, J. P. Ruurda, R. H. A. Verhoeven, R. van Hillegersberg

**Affiliations:** 1 Department of Surgery, University Medical Center Utrecht, G04.228, PO 85500, 3508 GA Utrecht, The Netherlands; 2 Netherlands Cancer Registry, Netherlands Comprehensive Cancer Organisation (IKNL), PO 19079, 3501 DB Utrecht, The Netherlands

**Keywords:** Gastric cancer, Minimally invasive, Survival, Lymph nodes, Learning curve

## Abstract

**Background:**

Minimally invasive techniques for gastric cancer surgery have recently been introduced in the Netherlands, based on a proctoring program. The aim of this population-based cohort study was to evaluate the short-term oncological outcomes of minimally invasive gastrectomy (MIG) during its introduction in the Netherlands.

**Methods:**

The Netherlands Cancer Registry identified all patients with gastric adenocarcinoma who underwent gastrectomy with curative intent between 2010 and 2014. Multivariable analysis was performed to compare MIG and open gastrectomy (OG) on lymph node yield (≥15), R0 resection rate, and 1-year overall survival. The pooled learning curve per center of MIG was evaluated by groups of five subsequent procedures.

**Results:**

Between 2010 and 2014, a total of 277 (14%) patients underwent MIG and 1633 (86%) patients underwent OG. During this period, the use of MIG and neoadjuvant chemotherapy increased from 4% to 39% (*p* < 0.001) and from 47% to 62% (*p* < 0.001), respectively. The median lymph node yield increased from 12 to 20 (*p* < 0.001), and the R0 resection rate remained stable, from 86% to 91% (*p* = 0.080). MIG and OG had a comparable lymph node yield (OR, 1.01; 95% CI, 0.75–1.36), R0 resection rate (OR, 0.86; 95% CI, 0.54–1.37), and 1-year overall survival (HR, 0.99; 95% CI, 0.75–1.32). A pooled learning curve of ten procedures was demonstrated for MIG, after which the conversion rate (13%–2%; *p* = 0.001) and lymph node yield were at a desired level (18–21; *p* = 0.045).

**Conclusion:**

With a proctoring program, the introduction of minimally invasive gastrectomy in Western countries is feasible and can be performed safely.

## Introduction

Since its introduction in 1994, minimally invasive gastrectomy (MIG) has been increasingly performed for gastric cancer surgery worldwide [1]. The possible advantages of minimally invasive surgery are diminished blood loss, shorter hospitalization, and reduced morbidity, at the cost of longer operation time [2, 3].

Several studies have compared MIG versus open gastrectomy (OG), demonstrating comparable short-term oncological outcomes [2, 3]. However, these studies were predominantly single-center studies conducted in the Asian population, in which patient and tumor characteristics differ from the Western population [4, 5]. The results of these studies are therefore difficult to extrapolate to the Western population.

In the Netherlands, MIG has been increasingly adopted after the introduction of a proctoring program. Since 2010, when only 4% of procedures performed was minimally invasively, the uptake has increased to 43% in 2014 [6]. It is however unclear if, during the early introduction of MIG, the short-term oncological outcomes were guaranteed. In this population-based cohort study, the feasibility of MIG regarding short-term oncological outcomes was evaluated during its introduction in the Netherlands.

## Materials and methods

### Patients

All patients who underwent a curative gastrectomy for adenocarcinoma of the stomach or gastroesophageal junction between 2010 and 2014 were included from the Netherlands Cancer Registry (NCR). Curative gastrectomies were defined as a gastrectomy for resectable tumors (pT1–4a) without metastatic disease (pM0) according to the 7th American Joint Committee on Cancer (AJCC) TNM gastric cancer staging system [7]. All patients had at least 1 year of follow-up. The NCR uses the national automated pathological archive (PALGA) as notification for all new malignancies in the Netherlands. Certified data managers of the NCR routinely extract information on patient and tumor characteristics from the medical records. Survival status is updated yearly from the civil registry. Intraoperative and clinical data are not routinely registered. The completeness of data registration is estimated to be high.

### Diagnostics and treatment

Diagnostic workup and treatment of patients were performed according to national guidelines [8]. In general, patients underwent staging with gastroscopy and tumor biopsy, followed by computed tomography (CT) of the thorax and abdomen. Because diagnostic laparoscopy was only recently included in the national guidelines (July 2016) [9], it was not performed routinely during the study period.

All fit patients with an advanced tumor (cT2+ N+) were offered a perioperative chemotherapy regimen similar or comparable to the MAGIC trial [10]. Perioperative radiotherapy was not routinely performed, except for some patients who received adjuvant chemoradiation as part of the CRITICS trial [11]. Surgery consisted of a partial or total gastrectomy, depending on the possibility to achieve an adequate proximal resection margin (≥6 cm) [8]. National guidelines recommend a D2 lymphadenectomy without station 10 dissection, pancreatectomy, and splenectomy. The choice for MIG or OG was based on the preferences of the hospital and surgeon. During the study period, gastric cancer surgery was centralized in the Netherlands, aiming at a yearly minimum of 20 resections per center. As a result, the number of centers performing gastrectomies was reduced from 35 centers in 2010 to 27 centers in 2014 [6]. All centers were included in this study, regardless of their previous experience.

Follow-up of patients consisted of medical history and physical examination at the outpatient clinic after 6 weeks, 6 months, 12 months, and yearly thereafter, until discharge of follow-up after 5 years. Radiologic imaging was not routinely performed during follow-up.

### Outcomes

Patient characteristics (age, gender, malignancy history), treatment characteristics (year of surgery, neoadjuvant treatment, extent of surgery), postoperative characteristics (hospital stay, in-hospital mortality, 90-day mortality), and tumor-specific characteristics (TNM stage) were included. For the analysis, all patients were divided into two groups according to the surgical procedure (MIG or OG). Short-term oncological outcomes were defined as lymph node yield, R0 resection rate, and 1-year overall survival. To identify a learning curve of MIG per center, the first 25 minimally invasive procedures were clustered per center, ranked, and pooled for all centers together. Subsequently, all procedures were divided into six groups (procedure 1–5, 6–10, 11–15, 16–20, 21–24,  >25) and compared for the conversion rate, radical resection rate, and lymph node yield.

### Statistical analysis

Data were analyzed using the IBM SPSS Statistics Version 20 for Windows and were considered significant if *p* < 0.05. Differences between MIG and OG in patient and tumor characteristics were analyzed with the chi-square test for ordinal variables. Continuous data were checked for normality and analyzed with the Student’s *t* test or one-way analysis of variance (ANOVA) for normally distributed data, and the Mann–Whitney *U* test or Kruskall–Wallis test for nonnormally distributed data. Lymph node yield was dichotomized with a cutoff value of 15 lymph nodes because it is a surgical quality indicator in the Netherlands [6]. Multivariable logistic regression was used to analyze lymph node yield (≥15) and R0 resection rates. Multivariable Cox regression was used to analyze the 1-year overall survival. Before performing the multivariable analyses, multiple imputation was performed for the missing values. After multiple imputation, missing pN stage was calculated from the number of positive lymph nodes according to the 7th AJCC TNM gastric cancer staging system [7]. Last, the pooled learning curve of MIG was analyzed by comparing the groups of five ranked procedures by one-way ANOVA or Kruskall–Wallis test after checking the normality of the data.

## Results

### Patient characteristics

**Fig. 1:**
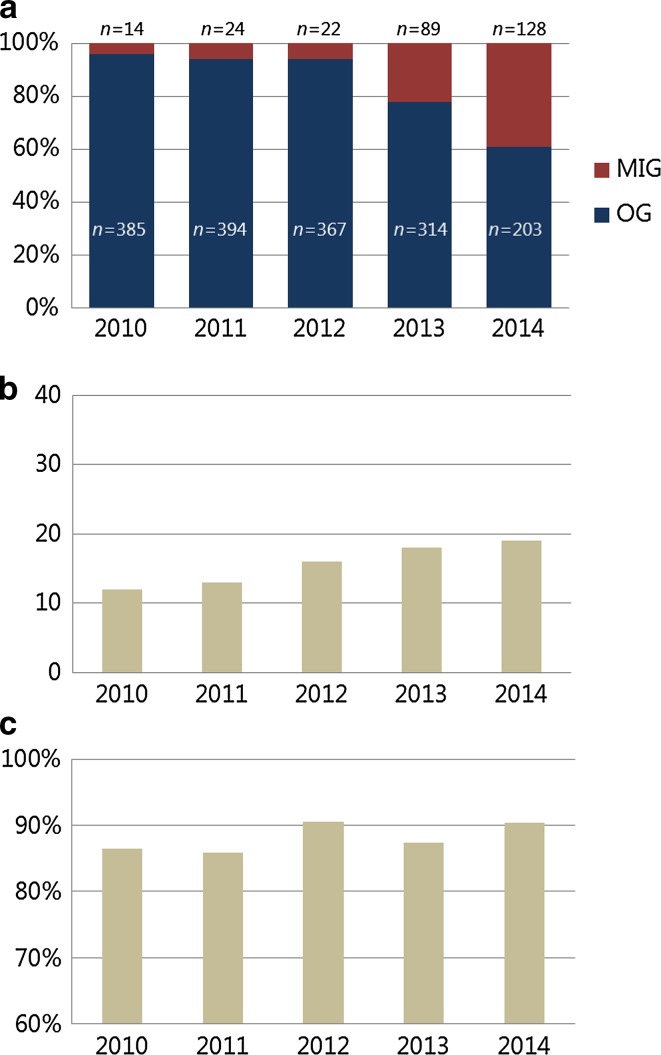
Change in minimally invasive gastrectomy (*MIG*) procedures (**a**), lymph node yield (**b**), and R0 resection rate (**c**) from 2010 to 2014. Total number of procedures per year was 399 in 2010, 418 in 2011, 389 in 2012, 403 in 2013, and 331 in 2014. *OG* open gastrectomy

A total of 1983 patients were included in this study. Data were missing for pT-stage (*n* = 17), radicality (*n* = 42), lymph node yield (*n* = 76), and number of positive lymph nodes (*n* = 41). Furthermore, the surgical approach was unknown for 43 patients. The remaining 1940 patients underwent OG in 1663 cases (86%) and MIG in 277 cases (14%). The baseline characteristics of these patients are presented in Table 1. Patients in the MIG group more often underwent total gastrectomy (*p* < 0.001), and more frequently received neoadjuvant (*p* < 0.001) or adjuvant treatment (*p* = 0.002), compared to patients in the OG. From 2010 to 2014, the percentage of patients who underwent MIG increased from 4% to 39% (*p* < 0.001; Fig. 1a), neoadjuvant chemotherapy increased from 47% to 62% (*p* < 0.001), and total gastrectomies increased from 29% to 40% (*p* = 0.001).

**Table 1:** Baseline characteristics of patients undergoing open gastrectomy (OG) and minimally invasive gastrectomy (MIG) for gastric adenocarcinoma with curative intent in the Netherlands from 2010 to 2014

	Open		Laparoscopy		*p*
	*n* = 1663	%	*n* = 277	%	
Age at diagnosis (years) [mean (± SD)]	68.4	[11.9]	68.5	[11.5]	0.961
Gender					0.935
Male	1035	(62)	173	(63)	
Female	628	(38)	104	(37)	
Malignancy history	202	(12)	30	(11)	0.516
Neoadjuvant treatment	858	(52)	175	(63)	<0.001
Chemotherapy	844	(51)	170	(61)	
Chemoradiotherapy	12	(<1)	4	(1)	
Radiotherapy	2	(<1)	1	(<1)	
Tumor location					<0.001
Proximal (cardia/fundus/corpus)	450	(27)	114	(41)	
Distal (antrum/pylorus)	746	(45)	103	(37)	
Overlapping	304	(18)	44	(16)	
Not specified	163	(10)	16	(6)	
Resection					<0.001
Partial	1109	(67)	140	(51)	
Total	554	(33)	137	(49)	
Conversions	–	–	24	(9)	
pT stage					0.370
T0	70	(4)	16	(6)	
T1–2	609	(37)	105	(39)	
T3–4	972	(59)	152	(56)	
Tx	12		4		
pN stage					0.691
N0	827	(51)	143	(52)	
N+	798	(49)	131	(48)	
Nx	38		3		
Tumor differentiation					0.553
Well–moderate	336	(20)	63	(23)	
Poor	787	(47)	123	(44)	
Unknown	540	(33)	186	(33)	
In-hospital mortality	79	(5)	13	(5)	0.701
<90-day mortality	128	(8)	17	(6)	0.404
Hospital stay (days) [median (range)]	10	[2–377]	8	[1–94]	<0.001
Adjuvant treatment	533	(32)	120	(43)	<0.001
Chemotherapy	408	(25)	97	(35)	
Chemoradiotherapy	122	(7)	23	(8)	
Radiotherapy	3	(<1)	0	(0)

### Lymph node yield

The median number of harvested lymph nodes was 16 (range, 0–39): 18 (range, 0–38) after MIG and 15 (range, 0–36) after OG. From 2010 to 2014 the lymph node yield increased from 12 (range, 0–39) to 20 (range, 0–39) (*p* < 0.001, Fig. 1b). Although univariable analysis demonstrated that MIG resulted in a high lymph node yield (≥15 nodes) compared to OG (OR, 1.63; 95% CI, 1.25–2.14; *p* < 0.001), in multivariable analysis this difference disappeared (OR, 1.01; 95% CI, 0.75–1.36; *p* = 0.944). Factors associated with a lymph node yield ≥15 were age younger than 65 years, a more recent year of surgery, neoadjuvant treatment, total gastrectomy, and a higher pTN stage (Table 2).

**Table 2:** Multivariable analysis of influence of MIG versus OG on the lymph nodes yield (≥15 nodes), R+ resection rate and 1-year overall survival in patients undergoing gastrectomy for gastric cancer

	Lymph node yield ≥15						R+ resection rate						1-year overall survival					
	Univariable			Multivariable			Univariable			Multivariable			Univariable			Multivariable		
	OR	[95% CI]	*p*	OR	[95% CI]	*p*	OR	[95% CI]	*p*	OR	[95% CI]	*p*	HR	[95% CI]	*p*	HR	[95% CI]	*p*
MIG	**1.63**	**[1.25–2.14]**	**<0.001**	1.01	[0.75–1.36]	0.944	0.74	[0.48–1.12]	0.156	0.86	[0.54–1.37]	0.523	1.01	[0.77–1.32]	0.969	0.99	[0.75–1.32]	0.962
Age ≥65	**0.63**	**[0.52–0.77]**	**<0.001**	**0.76**	**[0.61–0.95]**	**0.015**	1.01	[0.76–1.36]	0.936	-	-	-	**1.61**	**[1.29–2.01]**	**<0.001**	**1.50**	**[1.18–1.91]**	**0.001**
Female gender	1.05	[0.88–1.27]	0.579	-	-	-	1.25	[0.95–1.65]	0.108	1.30	[0.97–1.73]	0.080	1.19	[0.90–1.57]	0.219	-	-	-
Malignancy in history	**0.72**	**[0.55–0.95]**	**0.018**	0.79	[0.59–1.05]	0.109	1.20	[0.82–1.78]	0.352	-	-	-	1.29	[0.98–1.71]	0.123	1.12	[0.97–1.29]	0.420
Recent year of surgery	**1.39**	**[1.30–1.49]**	**<0.001**	**1.39**	**[1.29–1.49]**	**<0.001**	0.91	[0.83–1.00]	0.060	**0.89**	**[0.80–1.00]** ^*^	**0.047**	0.97	[0.91–1.04]	0.393	-	-	-
Neoadjuvant treatment	**1.79**	**[1.49–2.15]**	**<0.001**	**1.41**	**[1.15–1.76]**	**0.001**	0.94	[0.72–1.23]	0.663	-	-	-	**0.63**	**[0.52–0.76]**	**<0.001**	**0.69**	**[0.55–0.85]**	**0.001**
Total gastrectomy	**1.81**	**[1.49–2.20]**	**<0.001**	**1.46**	**[1.18–1.79]**	**<0.001**	**1.45**	**[1.10–1.91]**	**0.009**	1.25	[0.93–1.68]	0.134	**1.45**	**[1.19–1.75]**	**<0.001**	**1.54**	**[1.26–1.89]**	**<0.001**
pT3-4 stage	**1.44**	**[1.20–1.72]**	**<0.001**	**1.26**	**[1.01–1.56]**	**0.038**	**8.52**	**[5.42–13.40]**	**<0.001**	**5.80**	**[3.61–9.31]**	**<0.001**	**2.95**	**[2.34–3.73]**	**<0.001**	**1.90**	**[1.47–2.44]**	**<0.001**
pN+ stage	**1.50**	**[1.25–1.79]**	**<0.001**	**1.30**	**[1.06–1.60]**	**0.013**	**4.18**	**[3.04–5.75]**	**<0.001**	**2.10**	**[1.32–3.32]**	**<0.001**	**3.00**	**[2.42–3.71]**	**<0.001**	**2.29**	**[1.82–2.88]**	**<0.001**
Poor differentiation	**1.42**	**[1.12–1.80]**	**<0.001**	1.19	[0.92–1.53]	0.180	**2.73**	**[1.75–4.26]**	**<0.001**	**2.01**	**[1.32–3.32]**	**0.002**	**1.38**	**[1.08–1.77]**	**<0.001**	1.26	[0.98–1.62]	0.072

Analyses were performed using logistic regression (lymph node yield and R+ resection rate), and Cox regression (1-year overall survival). *Bold values* indicate statistically significant results (e.g. *p* < 0.05). Variables with *p* < 0.15 from univariable analysis and surgical approach were used for multivariable analysis

*OR* odds ratio, *HR* hazard ratio, *NA* not applicable

*p = 0.047

### Radicality

The R0 resection rate of all the procedures combined was 88%: 90% after MIG and 87% after OG. From 2010 to 2014, the R0 resection rate remained stable between 86% and 91% (*p* = 0.080; Fig. 1c). Both univariable and multivariable analysis demonstrated that the risk for an nonradical resection (R+) after MIG was comparable to OG (multivariable analysis: OR, 0.86; 95% CI, 0.54–1.37; *p* = 0.523) (Table 2). Factors associated with a R+ resection were surgery in earlier years, a higher pT or pN stage, and poor tumor differentiation.

### Survival

**Fig. 2:**
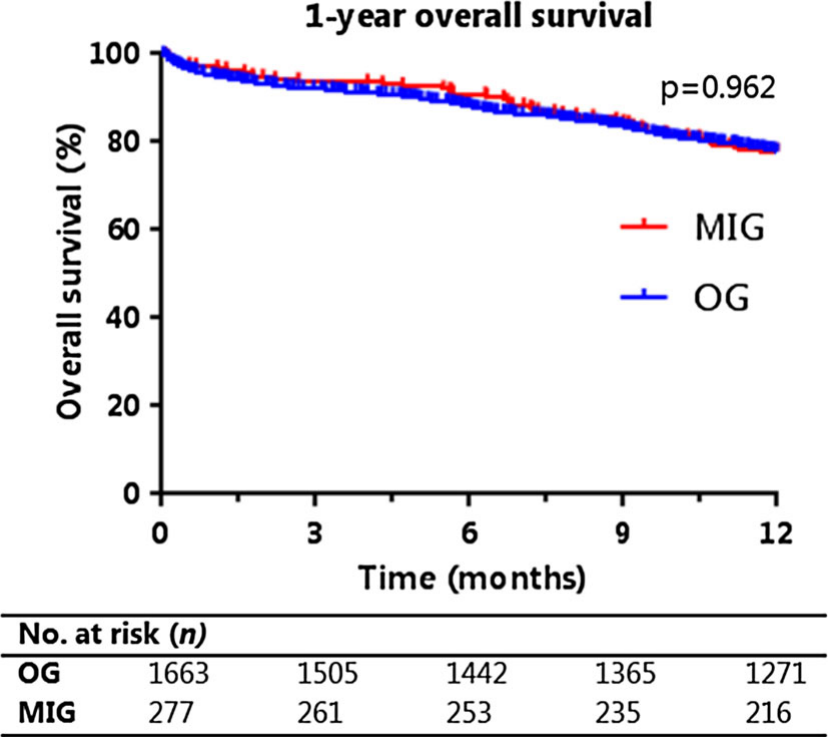
Kaplan–Meier curve of 1-year overall survival after MIG and OG

The 1-year overall survival of all patients was 78% and was also 78% after both MIG and OG. Kaplan–Meier curves of the 1-year overall survival are presented in Fig. 2. Both univariable and multivariable analysis demonstrated that the 1-year overall survival of MIG and OG were comparable (multivariable analysis: HR, 0.99; 95% CI, 0.75–1.32; *p* = 0.962) (Table 2). Factors associated with a prolonged survival were age younger than 65 years, neoadjuvant treatment, partial gastrectomy, and lower pT or pN stage.

### Learning curve

**Fig. 3:**
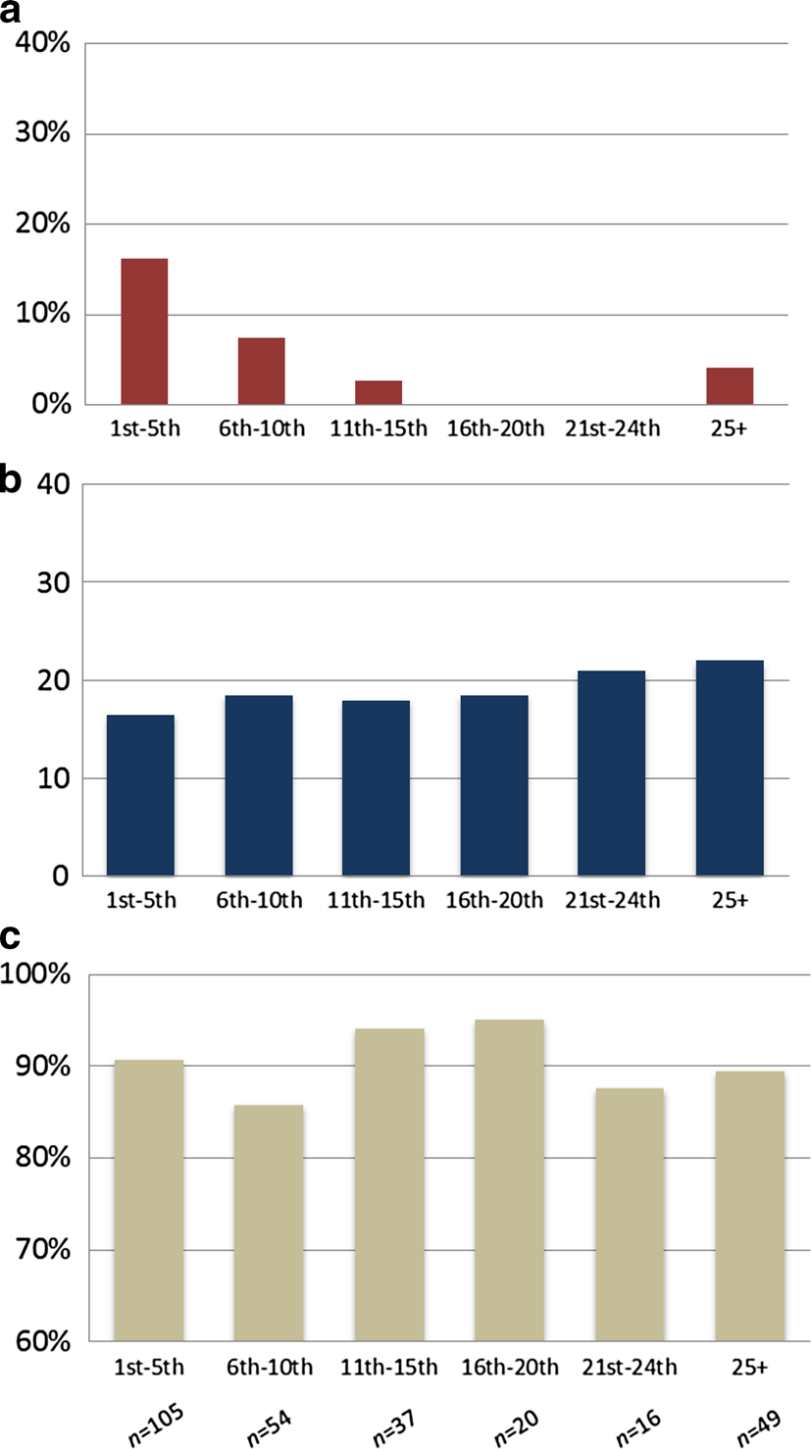
Pooled learning curve of MIG in the Netherlands for conversion rate (**a**), lymph node yield (**b**), and R0-resection rate (**c**). *Horizontal axis* represents number of MIG procedures per center. Total number of procedures per group was 105 (1th–5th), 54 (6th–10th), 37 (11th–15th), 20 (16th–20th), 16 (21st–24th), and 49 (≥25)

During the study period, a total of 29 centers performed at least 1 MIG procedure and only 4 centers performed 20 or more MIG procedures. After pooling all MIG cases and making groups of 5 cases each, 105 cases were classified as the first 5 procedures of all centers. The following groups consisted of 54 (6th–10th procedure), 37 (procedure 11th–15th), 20 (16th–20th procedure), 16 (21st–24th procedure), and 49 (≥25 procedures) cases. Figure 3 shows the conversion rates, lymph node yield, and radical resection (R0) rates per pooled group. After 10 procedures, the conversion rate decreased from 13% to 2% (*p* = 0.001), and the lymph node yield increased from 18 to 21 nodes (*p* = 0.045). No pooled learning curve could be demonstrated for the R0 resection rate.

## Discussion

This population-based cohort study is the first study on such a scale investigating the safety and feasibility of MIG regarding short-term oncological outcomes during the introduction in the West. The results demonstrate that during the introductory period of MIG in the Netherlands the lymph node yield, R0 resection rate, and 1-year overall survival were comparable to OG. Furthermore, a pooled learning curve of MIG was demonstrated in a decreasing conversion rate and an increased lymph node yield after 10 procedures, following an introduction with a structured proctoring program consisting of an introduction hands-on course and on-site proctoring.

In Asia, previous studies already demonstrated that the short-term oncological outcomes of MIG are comparable to OG [2, 3]. However, the Asian population consists of younger patients with lower tumor stages compared to Western populations [4]. These results are, therefore, difficult to extrapolate to Western countries. As this study is the first to evaluate the safety and feasibility of this procedure regarding short-term oncological outcomes on a large scale in a Western population, the results of the current study are relevant for all countries in the West.

There was a significant difference in the proportion of patients who received perioperative treatment and the extent of surgery between the MIG and OG group. As demonstrated by this study, these findings can be explained from a historical perspective: patients undergo (neo)adjuvant treatment and total gastrectomies more frequently in more recent years, whereas MIG is performed more often in more recent years as well. The increase in use of perioperative treatment can be contributed to the publication of the MAGIC trial [10], whereas the increase in total gastrectomies is most probably caused by the increase in gastroesophageal junction tumors [12]. To reduce the risk for confounding bias, these variables were included in the multivariable analysis comparing OG and MIG.

In addition to surgical approach, this study found other variables influencing short-term oncological outcomes, such as neoadjuvant chemotherapy. Surprisingly, the association between an increased lymph node yield and neoadjuvant chemotherapy is in contrast with reports in the literature [13, 14]. Other studies suggest that there is no difference or even a lower lymph node yield after neoadjuvant chemotherapy [13, 14]. Furthermore, this study found that a more recent year of surgery led to a higher lymph node yield. These findings might be explained by two developments in the Netherlands throughout recent years: centralization [15], and the nation-wide clinical audit (Dutch Upper-GI Cancer Audit, DUCA) for gastric cancer surgery [6]. Centralization of gastric cancer surgery in the Netherlands started in 2009, resulting in a decrease in hospitals performing fewer than 20 gastrectomies a year from 34 in 2011 to 16 in 2014 [6]. As centralization of gastric cancer surgery has been shown to improve short-term oncological outcomes, this can possibly explain the increase in lymph node yield and R0 resection rate found in this study [15–17]. Unfortunately, information on hospital volume could not be included in this study for reasons of the privacy restrictions of the Netherlands Cancer Registry. The DUCA was launched in 2011 and allowed hospitals to anonymously report the intraoperative and postoperative outcomes of gastric cancer surgery. This audit may have resulted in a higher awareness for lymph node yield and radical resections over the years, because these outcomes were seen as important parameters of adequate surgery.

This study demonstrated a pooled learning curve of MIG to be 10 cases. However, careful interpretation of the learning curve is warranted as these were performed through univariable analyses. Unfortunately, the privacy restrictions of the Netherlands Cancer Registry precluded using multivariable analysis for the learning curve. Furthermore, data on hospital volume or length of proctorship were unavailable. The demonstrated learning curve of 10 procedures is lower compared to an Asian study that demonstrated a learning curve in blood loss and operation time of 60 to 90 procedures [18]. This difference may be the result of several factors. First, we investigated the learning curve in conversion rates, lymph node yield, and radicality only. Other variables, such as blood loss, operation time, and complications, might have a different learning curve length. Second, we investigated a pooled learning curve per center instead of the learning curve of an individual surgeon. Last, most surgeons in the Netherlands who started MIG had experience in both open gastrectomy and laparoscopic surgery for other procedures. On the other hand, the difference could also indicate that Dutch surgeons in this study had not yet reached the plateau phase in their learning curve.

The pooled learning curve described in this study is after the University Medical Center Utrecht (UMC Utrecht) introduced this technique in the Netherlands in 2007 and set up a proctoring program. The UMC Utrecht invited other centers in the Netherlands to participate in the yearly organized “one-day course on minimally invasive gastrectomy.” In this hands-on course, participants receive lectures from experts and perform a minimally invasive gastrectomy on a cadaver together with an instructor. Furthermore, the UMC Utrecht offered centers to proctor their first MIGs in their own center. Because several centers were not involved in this training program, the pooled learning curve and surgical quality were not assessed in full but do reflect the daily practice in our country in this time frame. In the LOGICA-trial, the currently running Dutch multicenter randomized trial comparing MIG and OG, surgical quality has a key role. Before a center can participate in the trial it is proctored on site, has performed at least 20 MIGs, and should enable regular video and photo monitoring [19]. By these means, the trial aims to have a high surgical quality without the influence of a learning curve.

It is important to emphasize that the current study is a retrospective series with historical bias and learning curve bias. It solely gives an answer regarding the safety and feasibility of MIG in terms of short-term oncological outcomes during the introduction in the Netherlands, not whether OG and MIG are comparable in general. Unfortunately, no correction could be made on possible preoperative confounders, surgical or hospital volume, which were not available from the NCR. These variables and surgeon preferences could have possibly resulted in selection bias. Furthermore, this study was unable to analyze disease-free survival, as recurrence data also are not available from the NCR. In addition, this study did not analyze other relevant outcome measures of gastric cancer surgery such as intraoperative factors, morbidity, and quality of life. Thus, results from randomized controlled trials are necessary to make a fair comparison between MIG and OG. The only two large randomized controlled trials on this topic were conducted on distal gastrectomies in Asia and showed promising results for MIG [20, 21]. Current randomized controlled trials such as the LOGICA-trial, STOMACH-trial, and KLASS-trials are awaited to see if the promising results of MIG also account for total gastrectomy and in the West [19, 22, 23].

In conclusion, with a proctoring program, minimally invasive gastrectomy can be safely introduced regarding short-term oncological outcomes, and with a pooled learning curve of ten procedures for lymph node yield and conversion rate. Current randomized controlled trials should be awaited to determine if MIG is superior to OG.
